# High-Density EEG Source Localisation of averaged interictal epileptic Discharges validated by surgical Outcome

**DOI:** 10.1038/s41597-025-05740-z

**Published:** 2025-08-16

**Authors:** Bernd J. Vorderwülbecke, Margherita Carboni, Sebastien Tourbier, Laurent Spinelli, Denis Brunet, Martin Seeber, Christian M. Korff, Shahan Momjian, Maria Vargas, Margitta Seeck, Serge Vulliemoz, Jonathan Wirsich

**Affiliations:** 1 EEG and Epilepsy Unit, Division of Neurology, Geneva University Hospitals and University of Geneva, Rue Gabrielle-Perret-Gentil 4, 1205 Geneva, Switzerland; 2 Department of Neurology, Epilepsy-Center Berlin-Brandenburg, Charité – Universitätsmedizin Berlin, Charitéplatz 1, 10117 Berlin, Germany; 3 Functional Brain Mapping Lab, Department of Basic Neurosciences, University of Geneva, Campus Biotech, 9 Chemin des Mines, 1202 Geneva, Switzerland; 4 Connectomics Lab, Department of Radiology, Lausanne University Hospital, Rue du Bugnon 46, 1011 Lausanne, Switzerland; 5 CIBM Center for Biomedical Imaging, Geneva and Lausanne, Switzerland; 6 Neuropediatrics Unit, Geneva University Hospitals and University of Geneva, Rue Gabrielle-Perret-Gentil 4, 1205 Geneva, Switzerland; 7 Department of Neurosurgery, Geneva University Hospitals and University of Geneva, Rue Gabrielle-Perret-Gentil 4, 1205 Geneva, Switzerland; 8 Department of Neuroradiology, Geneva University Hospitals and University of Geneva, Rue Gabrielle-Perret-Gentil 4, 1205 Geneva, Switzerland

**Keywords:** Epilepsy, Electroencephalography - EEG, Predictive markers

## Abstract

Electroencephalographic source localisation (ESL) of interictal epileptiform discharges is a valuable tool for presurgical evaluation of pharmacoresistant focal epilepsy. Various forward models, inverse solutions algorithms, and software packages have been published. However, clinical validation studies are based on heterogenous end points and study cohorts. To allow comparison of different interictal ESL methods within one standardised dataset, we provide deidentified clinical data of 44 well-characterised patients with pharmacoresistant focal epilepsy and a first resective surgery, validated by 12-month postsurgical outcome. Thirty patients had favourable outcomes, including 28 with complete seizure freedom, indicating that the epileptogenic zone was correctly estimated. For each patient, pre-processed individual structural MRI, 257-channel EEG averages of homologous discharges, postsurgical structural neuroimaging, and detailed clinical and technical information are given. In patients with favourable outcomes, source maxima of averaged discharges were <10 mm remote from the resection in 67% and within a sublobe affected by the surgery in 83%. Future validation studies of new ESL approaches can be compared to this benchmark.

## Background & Summary

Six of 1,000 people worldwide suffer from epilepsy^1^, and a third do not become seizure-free despite the use of adequate antiseizure medication^2^. If pharmacoresistant epilepsy is focal, *i.e*., seizures are generated within networks limited to one brain hemisphere^3^, it can potentially be cured by surgical resection or disconnection of the specific brain regions that are indispensable for seizure generation: the ‘epileptogenic zone’. To delineate this conceptual zone as precisely as possible, surgical candidates undergo a thorough multimodal diagnostic evaluation including video-electroencephalographic (EEG) monitoring, structural magnetic resonance imaging (MRI), and neuropsychological testing. Further non-invasive and, if necessary, invasive diagnostic procedures are added according to the availability and expertise of the respective epilepsy centre^4,5^.

To increase the spatial accuracy of visual EEG interpretation, computer-based EEG source localisation (ESL) reconstructs the sources of scalp EEG activity within a 3D model of the brain^6,7^. ESL has mostly been applied on interictal epileptic EEG discharges (‘interictal ESL’). Interictal discharges can easily be marked and averaged^8^ whereas ESL of EEG seizure patterns (‘ictal ESL’) is technically challenging^9^. The diagnostic sensitivity of interictal ESL to correctly localise the epileptogenic zone is ~80%^10,11^.

Until now, the methodology of ESL is insufficiently standardised with a large variety of centre-specific analytic approaches. In principle, a biophysical forward model or ‘head model’ is needed to solve the forward problem: ‘Which scalp EEG voltage pattern results from a given intracerebral electric activity?’ and an inverse solution to solve the inverse problem: ‘Which intracerebral electric activity best explains a given scalp voltage EEG pattern?’^6,7,9^ A plethora of forward models exist, ranging from rather simple 3-shell sphere models to sophisticated realistic 7-compartment models^9^. Likewise, a variety of inverse models has been proposed and validated, including single-dipole models, linear distributed source models, sparse models, and beamformers. Commercial, certified software packages exist, besides freely available, open-source, and inhouse-manufactured software^7^. ESL can be based on high-density or low-density EEG recordings, on template heads, or individual MRI scans^6,7,9^.

In principle, each specific approach requires clinical validation, either by comparison of ESL results to the seizure onset zone as delineated by invasive EEG^12,13^ or by comparison to the ultimately resected brain area in relation to postsurgical seizure outcome^10^: If the source given by ESL is resected and the patient becomes seizure-free, ESL results can be considered a correct estimate of the epileptogenic zone. However, comparison across validation studies is difficult, since patient cohorts vary in size and type of epilepsy, definitions of concordance and of surgical outcome are heterogenous, and outcome is estimated at different time points after surgery^14–17^. While a few datasets of non-invasive and invasive EEG have been openly shared^18–20^, no dataset is publicly available that would allow comparison of various ESL approaches in the same patient cohort.

**Fig. 1:**
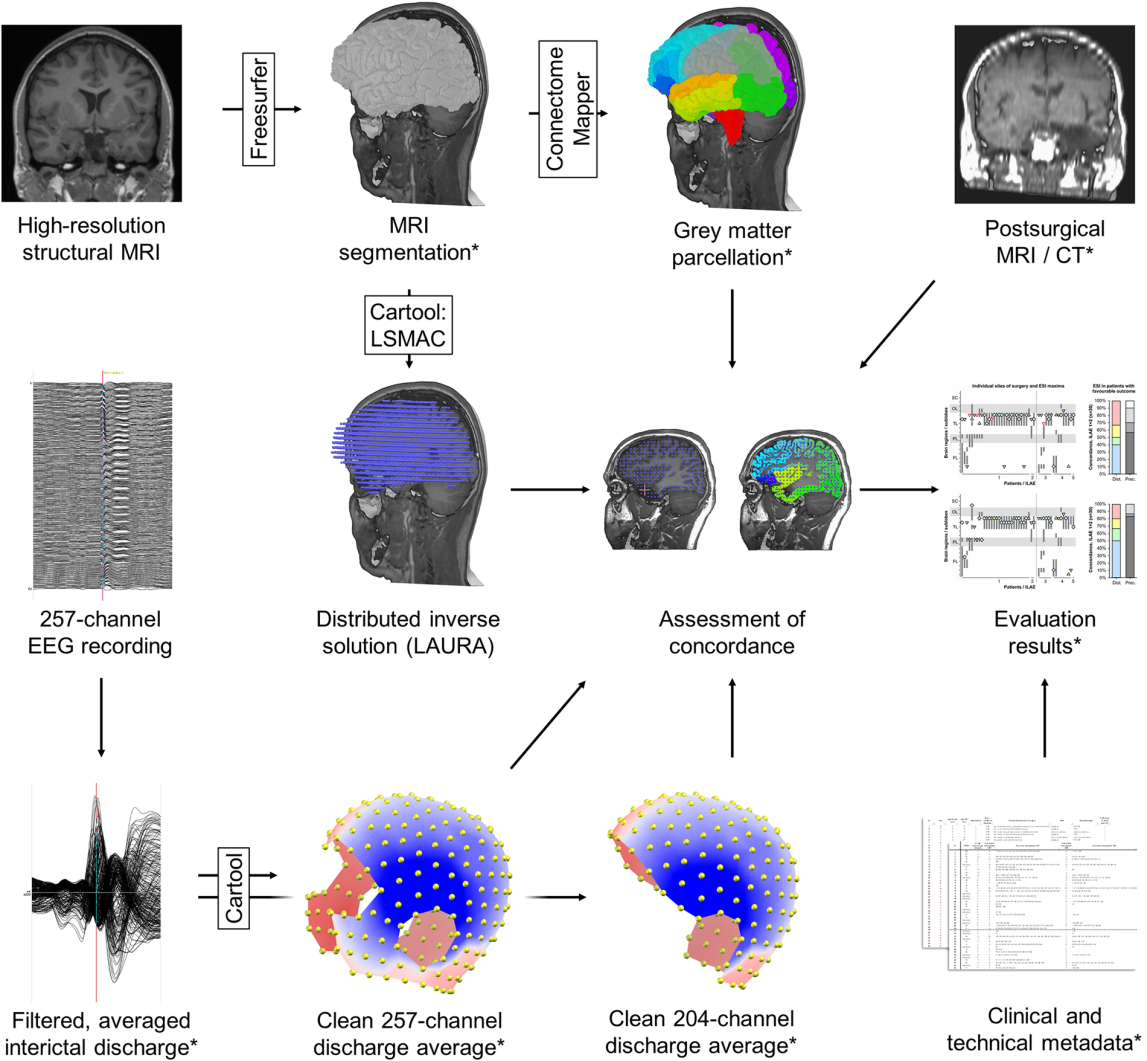
Overview of the processing and validation steps. Presurgical high-resolution T1 or MPRAGE was segmented using Freesurfer. The grey matter mask was parcelled using the Connectome Mapper 3. For the distributed inverse solution in Cartool, the Locally Spherical Model with Anatomical Constraints (LSMAC) served as head model and the Local Autoregressive Average (LAURA) as inverse solution. Interictal epileptic discharges were marked in 257-channel EEG recordings and averaged. 257-channel EEG was downsampled to 204 channels as an alternative approach, and corrupted channels were interpolated using Cartool. ESI accuracy was evaluated using postsurgical structural MRI or CT scan and 12-month postsurgical seizure outcome. Data marked with an asterisk (*) are made available in the dataset^21^. Figure modified from ref. ^22^.

To enable robust validation of interictal ESL using different forward models, inverse models, software packages, and EEG montages, we provide a large dataset obtained from 44 well-characterised patients with pharmacoresistant focal epilepsy^21^. Data include individual high-resolution presurgical MRI plus cortex parcellation, averaged interictal epileptic discharges recorded with 257-channel EEG, postsurgical MRI to allow comparison to the resected brain area, and detailed clinical information including 12-month postsurgical seizure outcome (Fig. 1). To date, two publications have been based on these data^17,22^. The first study showed that highest ESL accuracy was obtained by spatial down-sampling of the 257-channel EEG setup to 204 channels (compared to 257, 219, and 156 channels), and at 50% of the averaged discharge’s rising phase (compared to 10%, 25%, 75% and the peak)^17^. The second study revealed that among 6 linear distributed inverse solutions, the Low-Resolution Electromagnetic Tomography (LORETA) and the Local Autoregressive Average (LAURA) led to highest spatial accuracies^22^.

Because the data was collected primarily for clinical use and over the course of more than a decade, the dataset^21^ comes with some limitations. First, only averaged EEG data are available, with varying epoch duration and heterogenous filter settings. Second, the data were clinically validated only by concordance with the resected brain area and 12-month postsurgical outcome, but not by simultaneous or subsequent intracranial EEG recordings. Third, we provide interictal EEG data only while ictal data are considered even more relevant to delineate the epileptogenic zone. We encourage other researchers to provide further datasets without these drawbacks to enable even more profound evaluations of ESL pipelines.

## Methods

### Patients

Until July 31^st^, 2019, the database of the EEG and Epilepsy Unit at the University Hospitals of Geneva, Switzerland, was retrospectively screened for patients meeting the following criteria: (a) a first resective brain surgery to treat pharmacoresistant focal epilepsy; (b) age older than 6 years at evaluation; (c) presurgical 257-channel EEG recording with a minimum of 3 homologous focal interictal epileptic discharges; (d) presurgical high-resolution T1 or MPRAGE; (e) known 12-month postsurgical outcome according to the criteria of the International League Against Epilepsy (ILAE). ILAE 1 outcome implies complete seizure freedom since surgery (equivalent to Engel class IA), and ILAE 2 reflects occurrence of aware non-motor seizures (‘auras’) only (Engel IB). ILAE 1 and 2 were classified as favourable outcomes, whereas ILAE outcomes 3–6 indicate persistence of disabling seizures and were classified as unfavourable outcome^23^.

Among 304 patients screened, 45 patients operated between October 2007 and April 2018 fulfilled the inclusion criteria. In contrast to ref. ^17^, one case with extratemporal resection and ILAE 4 outcome (#39) was removed from the analysis^21^ because 257-channel ESI was performed after the initial epilepsy surgery. To assure transparency in respect to our previous studies, the patient is available in the database. Of the final 44 patients, thirty of these were female (68%), the median age at surgery was 19 years (range 7–53). Twenty-seven patients (61%) had temporal lobe epilepsy while 17 (39%) had extratemporal epilepsy, 34 (77%) had an MRI-visible epileptogenic lesion, 22 (50%) had additional intracranial EEG studies, and 30 (68%) had a favourable 12-month postsurgical seizure outcome. Demographic and clinical details on the study cohort are given in supplementary Table 1.

The work was carried out in accordance with the declaration of Helsinki and approved by the Cantonal Research Ethics Committee of Geneva (BASEC. 2020–02526). The ethics committee approved the reuse of these coded clinical data, including data from minors and including open data sharing. Patients were informed in a letter about the intended re-use of their data to be shared in coded form both nationally and internationally and were asked for their consent. Ten patients consented, and 35 did not reply or could not be reached. The data collected dated back till 2008 and as such not all patients could be reached for consent. In consequence, the ethics committee waived consent for those 35 participants that could not be reached or did not respond within 30 days.

### EEG acquisition and pre-processing

High-density EEG was recorded for the clinical purpose of presurgical epilepsy evaluation at the University Hospitals of Geneva using 257 electrodes (Philips EGI, now Magstim EGI, Eugene, US-OR). As part of the clinical routine, down-sampling and filtering were defined by the responsible EEG technician, and parameters were subject to change over time from 2007 to 2018. Therefore, provided EEG data have undergone heterogenous filtering and down-sampling (see supplementary Table 6). As part of the data archiving routine, raw EEG files were deleted after some time, so that unfiltered, raw data are no longer available.

EEG was recorded at sampling rates of 250–1000 Hz, unfiltered. High-density EEG was recorded continuously for 20 min to 20 h. EEG-experienced neurologists (M.S., S.V., and team) visually identified interictal discharges (<200 ms) using conventional montages of the 10–20 electrode array enhanced by 6 electrodes in the inferior temporal chain^24^. To avoid interference, discharges occurring earlier than 1 second after another were not considered. Discharges were clustered according to localisation and configuration. Before averaging, epochs were high-pass filtered at 0.3–1 Hz and low-pass filtered at 30–100 Hz (supplementary table 6). Per discharge cluster, 200-ms to 1000-ms epochs centred on the discharge’s peak were averaged using the most recent version of the Cartool software at that time (2007–2018)^6^. Information on the number of single discharges were not archived in 4 cases (supplementary Table 2).

Eight patients had more than one discharge cluster (supplementary Table 3). In these, the most frequently occurring cluster fitting with the electroclinical pattern was analysed. The known number of discharges per cluster ranged from 3–269 with a median of 28 (IQR 19–49), in line with the usual clinical experience. As an alternative to the full 257-channel setup, caudal electrodes covering cheeks and neck were removed using MATLAB R2016a (The MathWorks, Inc., Natick, US-MA), resulting in 204 EEG channels. Using Cartool 3.80 version 6164^25^, noisy channels were identified via visual inspection of both EEG waveforms and surface voltage maps, and information was interpolated from nearby channels using 3D splines. For the 257-channel setup, a median of 7 channels required interpolation (range 0–29) and for the 204-channel array, a median of 2 channels (range 0–20; supplementary table 2). In the case of EEG data available at 500–1000 Hz, the clean averages were temporally down-sampled to 250 Hz. In the end, the median number of timepoints across the averaged discharge’s rising slope was 11 (range 5–19; supplementary Table 2).

### MRI acquisition and pre-processing

During presurgical epilepsy evaluation, structural 3-Tesla cranial MRI was acquired with 0.7–1.0 mm slice thickness. T1 or MPRAGE images without contrast medium were re-sampled to 1 mm³ isotropic resolution through cubic interpolation using the Connectome Mapper 3 (CMP3) open-source pre-processing software^26,27^. As part of the CMP3, using Freesurfer version 6.0.1^28^, a grey matter mask was generated, excluding brainstem and cerebellum. With CMP3, the grey matter was parcelled into 82 regions of interest (41 per hemisphere) according to the ‘Lausanne parcellation’^29^ which is based on the Desikan-Killiany anatomical atlas^30,31^. The regions of interest were then grouped into 38 ‘sublobar’ areas (19 per hemisphere; supplementary table 4). Using Cartool, artefacts (hair, positioners, etc.) were removed from the whole-head MRI. As part of the clinical routine, postsurgical structural MRI was acquired around 3 months after surgery and linearly co-registered to the presurgical MRI using FSL5.0 (fsl-flirt)^32^. One patient (#14) had a postsurgical computed tomogram (CT) only which was linearly co-registered with SPM12 (‘coregister and reslice’ using mutual information, https://www.fil.ion.ucl.ac.uk/spm/software/, revision 7487). For deidentification purposes, postsurgical images were skull-stripped (FSL5.0/fslbet)^33^.

## Data Records

The dataset^21^ is freely available on the EBRAINS platform (10.25493/B3B8-XPM; registration and acceptance of terms of use required). The data is provided according to the brain imaging data structure (BIDS) specification^34^ (Table 1).

**Table 1:** Data structure description (BIDS derivative)^21^.

.	Root folder of Dataset
dataset_description.json	Meta data of dataset (authors, how to cite, etc.)
participants.tsv	Participant description
participants.json	Metadata of participant description
README	Readme (abstract of data descriptor)
CHANGES	Changelog of dataset
**/derivatives/cartool-v3.80/sub-patXX/ses-preop/eeg/**	Folder with averaged spike EEG data
sub-patXX_ses-preop_coordsystem.json	Definition of coordinate system (links to corresponding T1: sub-patXX_ses-preop_res-cmp_T1w.nii)
sub-patXX_ses-preop_desc-spikeerp-[204/257]c_channels.tsv	Channel description for EEG channels (257 channels and simplified layout of 204 channels)
sub-patXX_ses-preop_desc-spikeerp-[204/257]c_electrodes.tsv	Coordinates of electrodes (in mm and in the patient’s T1 space)
sub-patXX_ses_preop_task-rest_desc-spikeerp-257c_eeg.[vhdr/vmrk/eeg]	Averaged spike data provided in the BIDS-compatible BrainVision Core Data Format (vhdr/eeg/vmrk)
sub-patXX_ses_preop_task-rest_desc-spikeerp-[204c/257c]-interpol-250Hz_eeg.[vhdr/vmrk/eeg]	Processed averaged spike data provided in the BIDS-compatible BrainVision Core Data Format (vhdr/eeg/vmrk). Data was downsampled to 250Hz; noisy channels were interpolated from neighboring channels. All files are provided in a full 257-channel version and a reduced 204-channel version (electrodes covering cheeks and neck are removed).
**/derivatives/cmp-v3.0.0-beta-RC1/**	Folder containing all images treated with CMP3
atlas-L2018_desc-scale1_dseg.tsv	Names of the 82 regions of interest according to the ‘Lausanne parcellation’ (scale 1)
**/derivatives/cmp-v3.0.0-beta-RC1/sub-patXX/ses-preop/anat/**	Folder with preoperational structural MRI images
sub-patXX_ses-preop_res-cmp_T1w.nii.gz	Resampled T1-weighted acquisition to 1mm^3^ using CMP3 (electrode coordinates and post-operative images were coregistered to this space)
sub-patXX_ses-preop_T1w.json	BIDS-compatible metadata of original T1 acquisition
sub-patXX_ses-preop_res-cmp_desc-brain_T1w.nii.gz	Skull-stripped T1-weighted image
sub-patXX_ses-preop_res-cmp_desc-gm_mask.nii.gz	Binary Mask of segmented grey matter
sub-patXX_ses_preop_res-cmp_atlas-LS2008_desc-scale1_dseg.nii.gz	Grey matter parcellation into 82 regions of interest (41 per hemisphere) according to the ‘Lausanne parcellation’ (scale 1)
**/derivatives/cmp-v3.0.0-beta-RC1/sub-patXX/ses-postop/anat/**	Folder with preoperational structural MRI images
sub-patXX_ses-postop_space-individual_desc-brain_T1w.nii.gz	Skull-stripped post-operative image with resection co-registered to pre-operative T1 space
sub-patXX_ses-postop_space-individual_desc-brain_T1w.json	BIDS-compatible metadata of original post-operative T1 acquisition

BIDS, brain imaging data structure; CMP3, Connectome Mapper 3

To ensure de-identification of patient data in accordance with the ethics regulations while providing a valid skull surface for ESI, only pre-processed T1 data are provided instead of untreated raw data. The description of the originating T1 files is provided in brain imaging data structure (BIDS)^34^ json format. Using CMP3, MRI was converted to isometric 1mm^3^ resolution and parcellated (see above). Outputs for the MR images are described in the following section (available in folder /derivatives/cmp-v3.0.0-beta-RC1/sub-patXX/ses-preop). To deidentify the pre-processed head images, the viscerocranium outside the hd-EEG coverage was cut away using Cartool, as were the auricles as far as possible; eye areas were alienated.

In addition to the presurgical head image, the extracted brain (including brainstem and cerebellum) and the grey matter mask (without cerebellum; with and without parcellation) are provided (available in folder /derivatives/cmp-v3.0.0-beta-RC1/sub-patXX/ses-preop). In addition, the postsurgical segmented brains (FSL5.0/fslbet) derived from T1-head scans are provided, co-registered to the presurgical scans using FSL5.0/fslflirt^32^ (available in folder /derivatives/cmp-v3.0.0-beta-RC1/sub-patXX/ses-postop). Individual co-registered electrode coordinates are provided in tsv format both for 257 channels and 204 channels (coordinates are given in mm in the space of the provided presurgical T1). Averaged interictal epileptic discharges are provided in the BIDS-compatible BrainVision Core Data Format (vhdr/eeg/vmrk)^34^, (1) raw filtered average with 257 channels (sub-patXX_ses-preop_task-rest_desc-spikeerp-257c_eeg.eeg), (2) cleaned (noisy channels interpolated) and temporally down-sampled to 250 Hz with 257 channels (sub-patXX_ses-preop_task-rest_desc-spikeerp-257c_interpol_250 Hz_eeg.eeg), and (3) cleaned and temporally down-sampled to 250 Hz with 204 channels (sub-patXX_ses-preop_task-rest_desc-spikeerp-204c_interpol_250 Hz_eeg.eeg). All files are available in the folder /derivatives/cartool-v3.80/sub-patXX/ses-preop. In case no interpolation was carried out and the data of the clinical database was already 250Hz only one 257 channel file is provided and the ‘interpol’ keyword is omitted in the filename, information of interpolated channels can be found in Supplementary Table 2. If temporal down-sampling was unnecessary because the original data was already sampled at 250Hz (12 cases) or 125 Hz (one case), no ‘(…)desc-spikeerps-257c_eeg’ file is provided^21^. For details, see Supplementary Table 6.

## Technical and Clinical Validation

### Source reconstruction

The purpose of this clinical dataset^21^ is to enable validation studies of ESI algorithms and pipelines according to the needs and research plans of other scientists.

As a reference, we here provide results obtained with Cartool 3.80 version 6164^25^ previously published in ref. ^17^. Based on anatomical landmarks on the head surface, the average 257-electrode array was interactively co-registered to the patient’s individual 3D-MRI and later down-sampled to 204 channels. The Locally Spherical Model with Anatomical Constraints (LSMAC), a simplified realistic 3-shell head model, served as a forward model with consideration of age-adjusted skull thickness^6^. A regular 3D grid of approximately 5,000 sources was distributed throughout the grey matter mask. As an inverse model, the Local Autoregressive Average (LAURA) was used, a linear distributed inverse solution that imposes biophysical and physiological constraints on the minimum norm algorithm^35^. Data were filtered at [1:70] Hz with a 4^th^-order Butterworth filter to avoid phase distortion, plus notch filtering at 50 Hz. Using the default Tikhonov regularisation, the source of maximum amplitude at 50% of the averaged discharge’s rising phase was investigated.

### Comparison to site of resection and surgical outcome

In the following section, we summarize the approach of refs. ^17,22^ which is based on the distance between source maximum and resection cavity in relation to postsurgical seizure outcome. The patient’s postsurgical MRI or CT scan was co-registered to the solution point grid. The maximum source at 50% of the interictal epileptic discharge’s rising phase was visually compared to the resected brain area. Exact timepoints during the time course of the respective spike are displayed in supplementary Table 2. To allow comparison with other studies, two different measures of concordance were applied, distance to the edge of resection vs. level of precision. For distance, the Euclidian distance between the maximum source and the nearest border of resection was approximated by inspecting the 3 orthogonal planes (axial, sagittal, coronal). If the source was inside the resection cavity, the distance was considered 0 mm. Otherwise, the distance was classified as <10 mm, <20 mm, or >20 mm from the border of the resection. Distances of 0–10 mm were considered concordant. For the level of precision, the maximum source was compared to sublobes, lobe(s), and hemisphere affected by the surgery, derived from the ‘Lausanne parcellation’^29^ (supplementary Table 4). Sublobar precision was considered concordant^17,22^.

As previously described, concordance in patients with favourable 12-month surgical outcome (ILAE 1 + 2) was considered as true positive (TP), while discordance in unfavourable outcome patients (ILAE 3–5) was true negative (TN). Likewise, concordance in case of unfavourable outcome was considered as false positive (FP) and discordance in favourable outcome as false negative (FN). Sensitivity was calculated as TP / (TP + FN), and specificity as TN / (TN + FP). Overall accuracy was calculated as (TP + TN) / (TP + TN + FP + FN). The diagnostic odds ratio (OR) of favourable post-surgical outcome in case of concordant *vs*. discordant ESI results was calculated as (TP*TN) / (FP*FN)^10,17,22^.

**Fig. 2:**
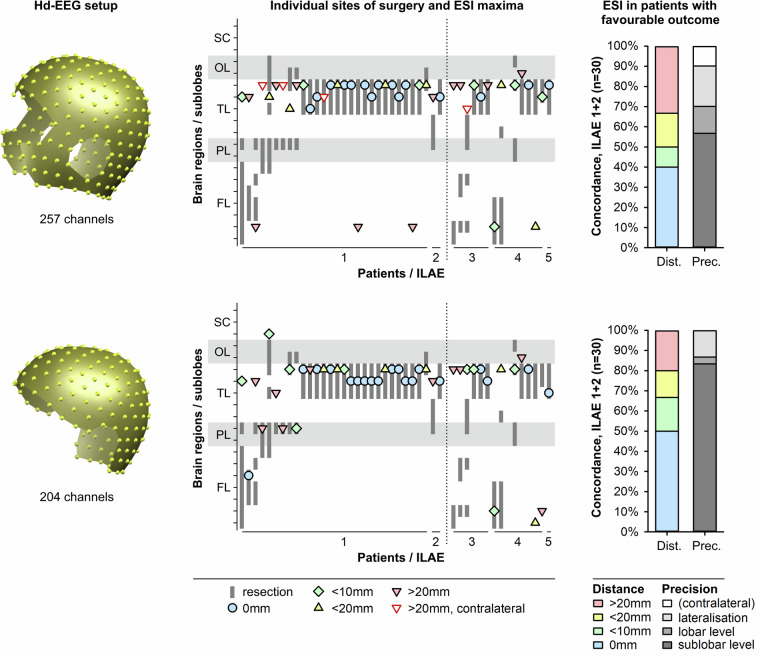
Validation of the setup using 257 EEG electrodes (above) and 204 electrodes (below). Left, 3D overview of the setup. Centre, individual sites of surgery, and ESI maxima obtained at 50% of the averaged interictal epileptic discharge’s rising phase. X-axis: Individual patients, sorted by 12-month outcome according to the ILAE classification. Y-axis: ESI maximum (symbol) compared to brain areas affected by resection (grey bar), given on a sublobar level, sorted by brain regions. SC, subcortical grey matter; OL, occipital lobe; TL, temporal lobe; PL, parietal lobe; FL, frontal lobe. Symbols indicate distances between ESI maximum and resected brain area. Right, overview of results in those 30 patients with favourable 12-month outcome only. Left coloured bar: distance between maximum source and resected brain area. Right greyscale bar: level of precision. Figure modified from ref. ^17^; for discussion of the results, see there.

Results of the validation using 257 and 204 EEG channels are detailed in Table 2 and Fig. 2 as previously published^17,22^. In contrast to ref. ^17^, one case with extratemporal resection and ILAE 4 outcome (#39) was removed from the analysis^21^ because 257-channel ESI was performed after the initial epilepsy surgery. To assure transparency in respect to our previous studies, the patient is available in the database. In summary, accuracy was higher with ESI based on 204 channels (59% and 73%) than on 257 channels (50%). Sensitivity, specificity, overall accuracy, and diagnostic odds ratio were higher for the sublobar precision approach than for the 0–10 mm distance approach. Sensitivity was higher in cases of temporal lobe epilepsy while specificity was higher in cases of extratemporal epilepsy. Results were homogenous across averages of less or more than 10 discharges or unknown numbers of single discharges^17^. Detailed individual ESI results in comparison to the resected brain areas are described in supplementary Table 5 as well as in ref. ^17^.

**Table 2:** Data validation based on 257 and 204 EEG channels, corrected from^17^.

	257 channels		204 channels	
	ESI measure:	ESI measure:	ESI measure:	ESI measure:
	distance 0–10 mm	sublobar precision	distance 0–10 mm	sublobar precision
**Concordance, all patients (positives, P)**	50% (22/44)	57% (25/44)	63% (28/44)	73% (32/44)
**Concordance, extratemporal resections (P)**	18% (3/17)	18% (3/17)	47% (8/17)	47% (8/17)
**Concordance, temporal resection (P)**	70% (19/27)	81% (22/27)	74% (20/27)	89% (24/27)
**Concordance, ILAE 1 + 2 (TP)**	50% (15/30)	57% (17/30)	67% (20/30)	83% (25/30)
**Concordance, ILAE 3–5 (FP)**	50% (7/14)	57% (8/14)	57% (8/14)	50% (7/14)
**Sensitivity, all patients**	50%	55%	67%	83%
**Sensitivity, extratemporal resections**	9%	9%	46%	55%
**Sensitivity, temporal resection**	73%	84%	79%	100%
**Specificity, all patients**	50%	38%	43%	50%
**Specificity, extratemporal resections**	67%	67%	50%	67%
**Specificity, temporal resection**	38%	25%	38%	38%
**Overall accuracy, all patients**	50%	50%	59%	73%
**Overall accuracy, extratemporal resections**	29%	29%	47%	58%
**Overall accuracy, temporal resection**	63%	67%	67%	82%
**Diagnostic OR, all patients**	1.0	0.8	1.5	5.0
**Diagnostic OR, extratemporal resections**	0.2	0.2	0.8	2.4
**Diagnostic OR, temporal resection**	1.7	1.8	2.3	∞

TP, true positives; FP, false positives; OR, odds ratio. In contrast to ref. ^17^, one case with extratemporal resection and ILAE 4 outcome (#39) was removed from the dataset^21^ because 257-channel ESI was performed after the initial epilepsy surgery.

## Usage Notes

EEG Data is provided in BIDS^34^ compatible eeg/vhdr BrainVision Core Data Format and easily importable with common EEG software such as EEGLab, Fieldtrip, Brainstorm, and SPM. Imaging data is provided in nii format. All data are pre-processed for anonymization purposes and stored as BIDS derivative^21^. The above-described results from refs. ^17,22^ can be used as a benchmark to evaluate new forward models and inverse solutions against distance from resection (supplementary Table 1) and surgery outcome (particpants.tsv and supplementary Table 1).

## Supplementary information

Supplementary Tables

## Data Availability

Connectome Mapper 3 (CMP3) is freely available at https://connectome-mapper-3.readthedocs.io. The Cartool software is freely available for use in public research and non-commercial purposes (http://cartoolcommunity.unige.ch, https://github.com/DenisBrunet/Cartool).
